# Editorial: Novel regulatory mechanisms behind thermogenesis of brown and beige adipocytes

**DOI:** 10.3389/fendo.2023.1268299

**Published:** 2023-08-18

**Authors:** Abhirup Shaw, Endre Kristóf, Rubén Cereijo

**Affiliations:** ^1^ Laboratory of Cell Biochemistry, Department of Biochemistry and Molecular Biology, Faculty of Medicine, University of Debrecen, Debrecen, Hungary; ^2^ Rosalind & Morris Goodman Cancer Institute, McGill University, Montreal, QC, Canada; ^3^ Departament de Bioquímica i Biomedicina Molecular, Institut de Biomedicina de la Universitat de Barcelona (IBUB), and Institut de Recerca de Sant Joan de Déu, Universitat de Barcelona, Barcelona, Spain; ^4^ CIBER Fisiopatología de la Obesidad y Nutrición, Madrid, Spain

**Keywords:** obesity, brown adipocytes, beige adipocytes, UCP1, thermogenesis, beiging, browning

## Introduction

Brown adipose tissue (BAT) has emerged as a pivotal organ in the field of metabolic research due to its unique properties. Unlike white adipose tissue (WAT), which predominantly stores energy in the form of triglycerides, BAT possesses a remarkable capacity to dissipate energy as heat through the process of non-shivering thermogenesis (1). This specialized function is primarily attributed to a high abundance of mitochondria and the expression of uncoupling protein (UCP) 1 within brown and beige adipocytes - a subpopulation of thermogenic cells that can originate from WAT depots in response to certain stimuli (1). Over the past decade, there has been a resurgence of interest in BAT as a potential therapeutic target for the rising global epidemic of obesity and related metabolic disorders, since the (re)discovery of active BAT in adult humans overturned the conventional belief that BAT exclusively existed in newborns to regulate body temperature (2). The identification and understanding of key regulators and molecular pathways governing BAT have provided exciting prospects for the development of new approaches to combat obesity and concomitant metabolic diseases (1, 2). However, despite such significant advancements, numerous questions regarding its precise regulatory mechanisms, interactions with other organs, and potential long-term effects on overall metabolic health remain unanswered. This Research Topic aimed to comprehensively integrate original studies and state-of-the-art knowledge on BAT and its physiological role in energy expenditure, as well as the underlying molecular pathways and potential therapeutic implications.

## Regulatory mechanisms in brown/beige adipocytes

Wang et al. comprehensively reviewed the transcriptional cascades, epigenetic modifications, non-coding RNAs, and endogenous or exogenous metabolites which can regulate brown/beige adipocyte differentiation from preadipocytes or white to beige transdifferentiation. The paracrine and endocrine factors that mediate the communication between either different cell types of BAT or distinct organs are also discussed.

Mitochondrial biogenesis, clearance, and dynamics are key processes in maintaining the proper functions of adipocytes (3). Brown/beige adipocytes convert the subtracted energy of nutrient molecules into heat (1, 4). In the mini review written by Chang, the regulation of how the heterogeneous mitochondrial populations of thermogenic adipocytes select distinct nutrient molecules was discussed. Moreover, Zheng et al. systematically reviewed how these processes contribute to the regulation of thermogenesis, browning, beige to white transition, and glucolipid metabolism. Furthermore, the potential applications of compounds present in herbal extracts in the treatment of obesity by the induction of mitochondrial functions of adipocytes are also summarized.

The circadian clock is maintained by self-sustained transcription-translation feedback loops by which organisms anticipate and adapt to the regular daily environmental cues, such as light (5). The circadian oscillation of clock genes also contribute to the regulation of adipocyte browning and thermogenesis reviewed by Peng and Chen. This is underlined by epidemiologic studies and laboratory interventions which provided evidence that reduced sleep duration and quality represent risk factors for the development of obesity and type 2 diabetes (6).

Metabolites generated from nutrients also contribute to the replenishment of vital macromolecules within adipocytes. Vámos et al. modelled thermogenic differentiation and conversion into the inactive state of human subcutaneous adipocytes. By global gene expression and metabolomics analyses, the critical importance of single nucleotide polymorphism at fat mass and obesity-associated *(FTO)* rs1421085 locus was revealed in the aforementioned processes most prominently by the regulation of several metabolic pathways, including amino acid utilization *via* alanine-serine-cysteine (ASC) transporter 1, which was postulated to support β-adrenergic-driven thermogenic activation (7).

On the other hand, proteostasis has also been shown to be a mechanism relevant in thermogenic adipocytes (8). In this regard, Koçberber et al. explored the role of two proteasome activators, PA28αβ (*Psme1*) and PA200 (*Psme4*), in brown adipocyte differentiation and function. Interestingly, siRNA-mediated studies revealed a dispensable role on murine brown adipocyte proteostasis, adipogenesis, and thermogenesis, prospecting further research on protein turnover complex regulation in BAT.

## Non-sympathetic molecular induction of beiging

Beyond classic β-adrenergic stimulation, some non-sympathetic mechanisms can physiologically regulate BAT functions (9). In this Research Topic, several authors also aimed to describe novel molecules capable of inducing human brown/beige adipocyte recruitment. By differentiating adipocytes from individuals with obesity, Coulter et al. showed that β–carotene can act synergistically with naringenin to induce expression of UCP1 and glucose metabolism-associated genes *in vitro*, as well as inducing lipase-mediated triglyceride hydrolysis in beige adipocytes. Ali et al. also demonstrated that another diet-derived bioactive compound, allicin, can also trigger a beigeing transcriptomic program in human adipocytes, with rearrangements in mitochondrial morphology and lipid droplet dynamics. Beyond dietary nutrients, Nagy et al. reported that nicotinamide-riboside, a NAD^+^ precursor, can also induce a respiration uncoupling-based beige phenotype both at the transcriptional and functional level in human primary adipocytes, a process mediated *via* sirtuin-1.

## Novel insights on the endocrine role of human BAT

Beyond its energy-dissipating functions, a secretory endocrine role has currently been well-established for BAT. Analogously to WAT-derived adipokines, BAT can communicate with distant organs by releasing so-called brown adipokines (or batokines) to orchestrate beneficial systemic metabolic actions (10). While this has been extensively explored in experimental models, batokine studies in humans are still scarce. In this topic, Garcia-Beltran et al. shed some light into this question by assessing expression and circulating levels of a batokine, meteorin-like (METRNL) (11), in human newborns. They demonstrated that METRNL is highly expressed in neonatal interscapular BAT, and that its circulating levels correlate to those of chemokine (C-X-C) motif ligand (CXCL) 14, another known batokine (12). Moreover, they were also associated with infrared-assessed BAT thermogenic activation, indicating METRNL is a new biomarker of BAT activity in early life.

## Closing remarks and future perspectives

The contributions to this Research Topic have remarkably bestowed to elucidate the intricate biology of BAT, adding novel insight to the growing body of evidence supporting its role in human metabolic homeostasis. While further research is needed, fully ascertaining the regulatory mechanisms ruling brown/beige adipocyte differentiation, especially in human models, will ultimately provide new avenues for therapeutic intervention against obesity and associated metabolic disorders in the pursuit of improved public health.

## Author contributions

AS: Writing – original draft, Writing – review & editing. EK: Writing – original draft, Writing – review & editing. RC: Writing – original draft, Writing – review & editing.
